# Afferent loop obstruction induced by gallstone ileus after Whipple procedure: a case report and literature review

**DOI:** 10.3389/fmed.2025.1522046

**Published:** 2025-03-05

**Authors:** Danfeng Shen, Haibin Xu, Peng Chang, Qiang Gu, Hongxing Xu

**Affiliations:** Department of Hepatobiliary Surgery, Taicang Affiliated Hospital of Soochow University, Suzhou, China

**Keywords:** afferent loop obstruction, gallstone ileus, Whipple procedure, internal hernia, pancreatojejunostomy

## Abstract

Afferent loop obstruction (ALO) induced by gallstone ileus after pancreaticoduodenectomy (PD) is rare. We present a case of ALO after the Whipple procedure in a 62-year-old male with a rare cause of gallstone ileus. He was admitted to the hospital with epigastric pain, fever, and jaundice two years after experiencing intermittent abdominal pain. With the help of computed tomography (CT) and magnetic resonance cholangiopancreatography (MRCP) gallstone ileus was confirmed and the patient underwent emergency surgery. Intraoperative findings indicated that internal herniation, adhesions and a stuck intra-pancreatic duct catheter caused gallstone formation and eventually led to ALO. The patient underwent prompt surgical intervention and ultimately recovered well. This case report holds a significant potential to contribute to the focus on the details of the Whipple procedure to prevent ALO and gallstone ileus.

## Introduction

ALO is a rare postoperative complication of PD that mainly results from tumor recurrence, adhesion or internal hernia (1). ALO can lead to serious conditions such as biliary tract infection, acute pancreatitis, intestinal necrosis or perforation, with high morbidity and mortality rates, often requiring emergency treatment. Gallstone ileus is a rare form of intestinal obstruction, defined as a small or large bowel obstruction mostly secondary to a biliary enteric fistula (2). It is extremely rare in postcholecystectomy patients (3).

Previous reports have shown small bowel obstruction caused by gallstones or bezoars after the pylorus preserving Whipple procedure and ALO caused by an enterolith after the Whipple procedure (3–5). In contrast, ALO induced by gallstone ileus after the Whipple procedure has rarely been reported. We report the case of a 62-year-old man who presented with ALO caused by a gallstone after 8 years after the Whipple procedure for cholangiocarcinoma. This study was reported in line with the SCARE criteria (6).

## Case presentation

**Figure 1:**
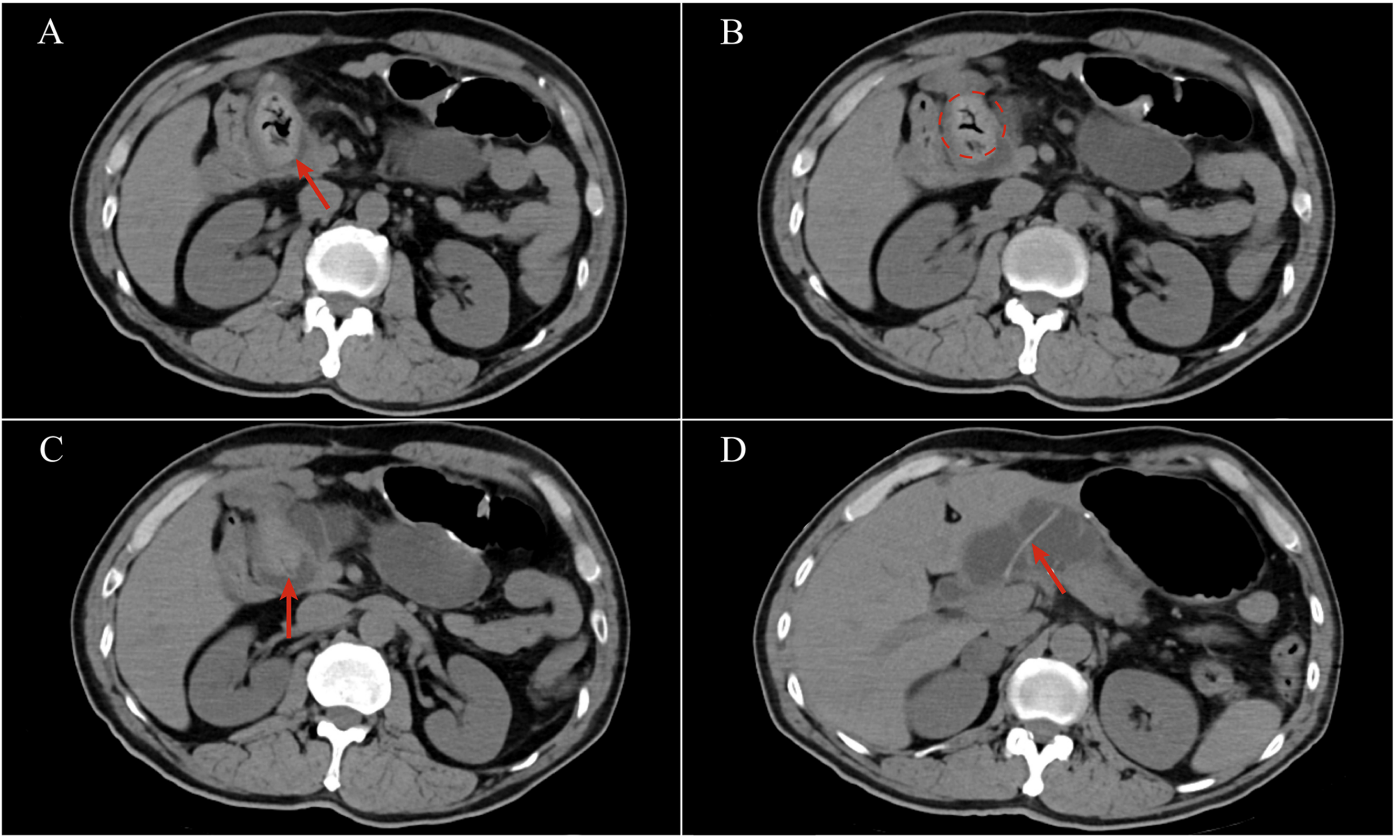
CT images: **(A)** An unidentified mass in the afferent loop with gas inside; **(B)** a “Mercedes-Benz sign”; **(C)** a catheter in the middle of the unidentified mass; **(D)** a catheter was stuck in the afferent loop.

**Figure 2:**
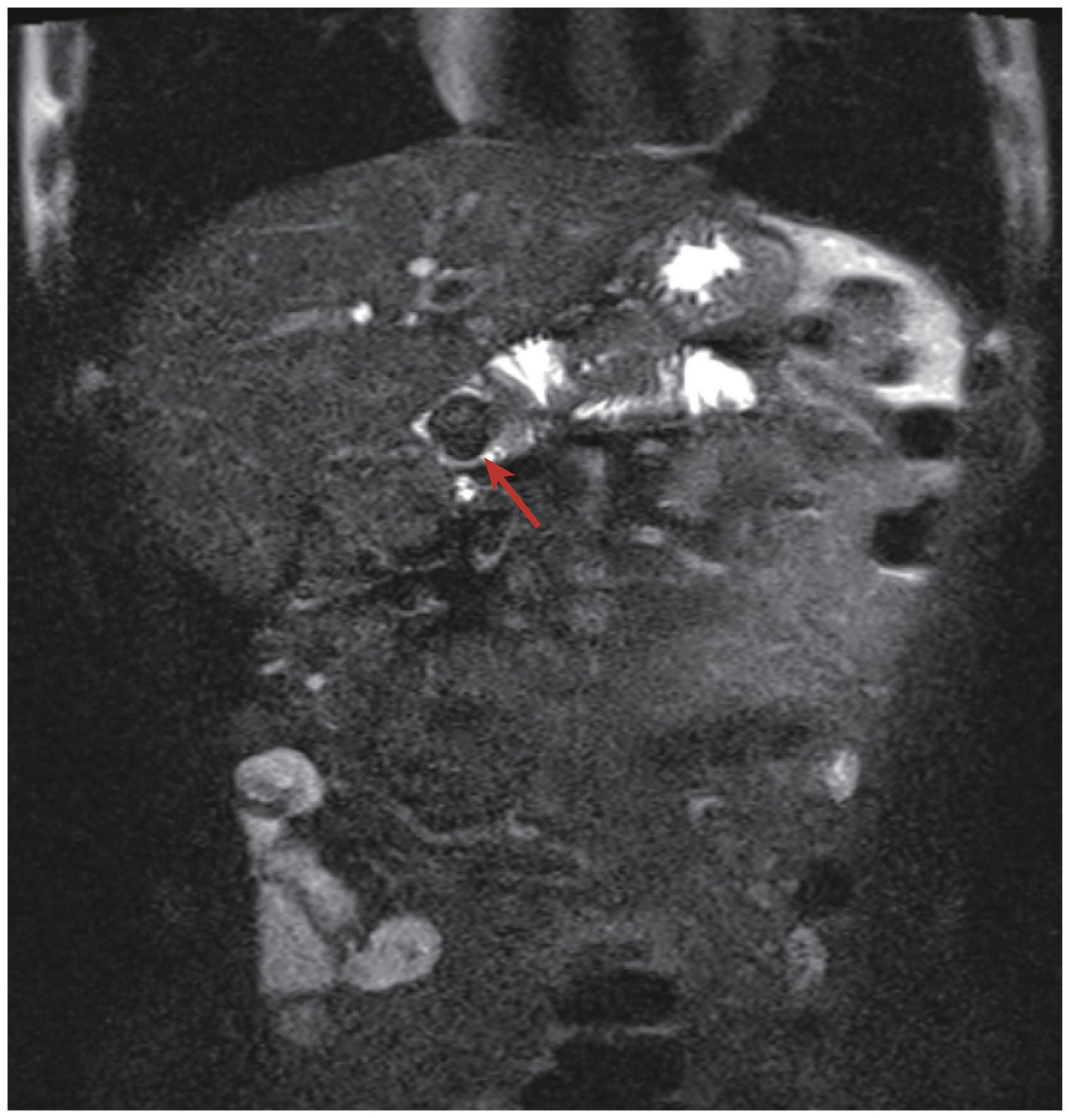
MRCP images: the gallstone ileus was observed (red arrow) and the afferent loop proximal to the obstruction was markedly dilated.

A 62-year-old male, admitted to our centre for increasing epigastric abdominal pain and high fever for 6 h. Vital signs were unremarkable, and physical examination revealed yellowing of the skin and sclera and marked epigastric tenderness. He underwent open PD (Whipple procedure) for cholangiocarcinoma 8 years ago. Postoperative pathology diagnosed cholangiocarcinoma (T1M0N0), which did not receive chemotherapy. Two years ago, he began to suffer from intermittent abdominal pain and did not receive any examination or treatment. He had a history of hypertension and diabetes mellitus with good blood pressure and glucose control. Laboratory tests revealed elevated white blood cell count (12.6 × 10^9^/L, normal range 4.0–10.0 × 10^9^/L), interleukin-6 (426.48 pg./mL, normal range 0.0–7.0 pg./mL), total bilirubin (52.0 umol/L, normal range 3.0–22.0 umol/L), aspartate transaminase (AST) (464.0 U/L, normal range 0.0–50.0 U/L), alanine aminotransferase (ALT) (230.0 U/L, normal range 0.0–40.0 U/L), serum amylase (AMY) (582.0 U/L, normal range 30.0–110.0 U/L) and carbohydrate antigen (CA) 19–9 (99.93 U/mL, normal range 0.0–37.0 U/mL). Emergency CT scan revealed bowel obstruction caused by an unidentified mass near the biliary-enteric anastomosis of the afferent loop (Figure 1). As the “Mercedes-Benz sign” was found in CT images, the unidentified mass was highly suspicious to be a gas-containing gallstone. Meanwhile, a slender tubular object was stucked in the afferent loop, suspected to be a intra-pancreatic duct catheter used for the pancreatic-enteric anastomosis during Whipple procedure. A stone-like object was observed and the afferent loop was markedly dilated in MRCP images. No significant intrahepatic biliary dilatation was observed on the CT and MRI images of this patient. Relying on the MRCP and CT images, a rare form of ALO induced by gallstone ileus after the Whipple procedure was finally confirmed (Figure 2). Consequently, the preoperative diagnosis was ALO, gallstone ileus, obstructive jaundice, secondary biliary tract infection and pancreatitis. To solve this problem, we performed an endoscopic procedure. However, gastroscopy was unable to reach the site of obstruction due to severe bowel twisting. We further attempted the CT-guided placement of an afferent loop decompression catheter. The interventional guidewire was barely able to reach the blockage, but could not pass through on multiple attempts.

**Figure 3:**
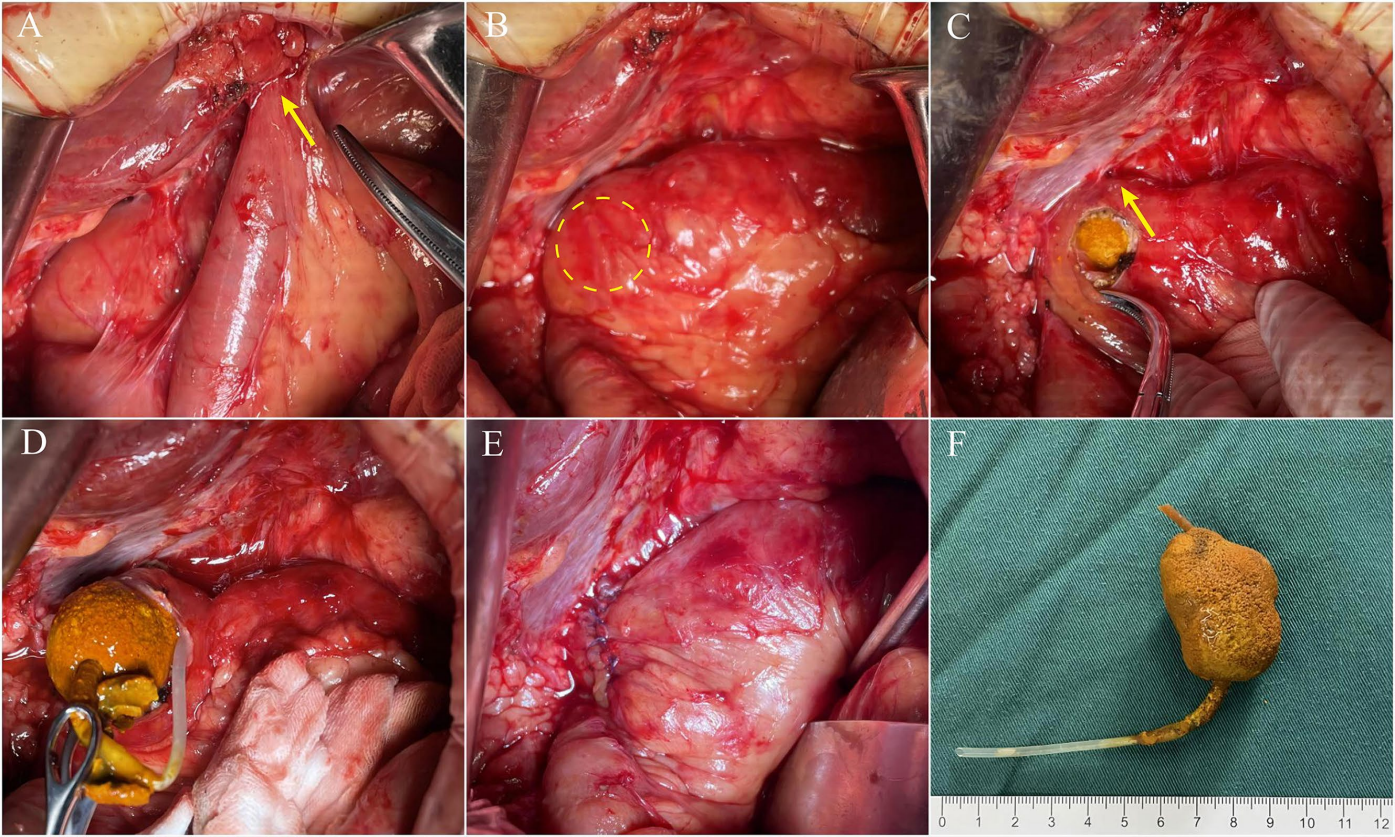
Intraoperative findings: **(A)** The afferent loop adhered to the liver and formed an acute angle (yellow arrow); **(B)** the site of the obstruction; **(C)** the longitudinal dissection of the intestine and the site of the biliary-enteric anastomosis (yellow arrow); **(D)** the catheter wrapped in the stone; **(E)** the closed afferent loop incision; **(F)** an “lollipop”: the removed gallstone and catheter.

**Figure 4:**
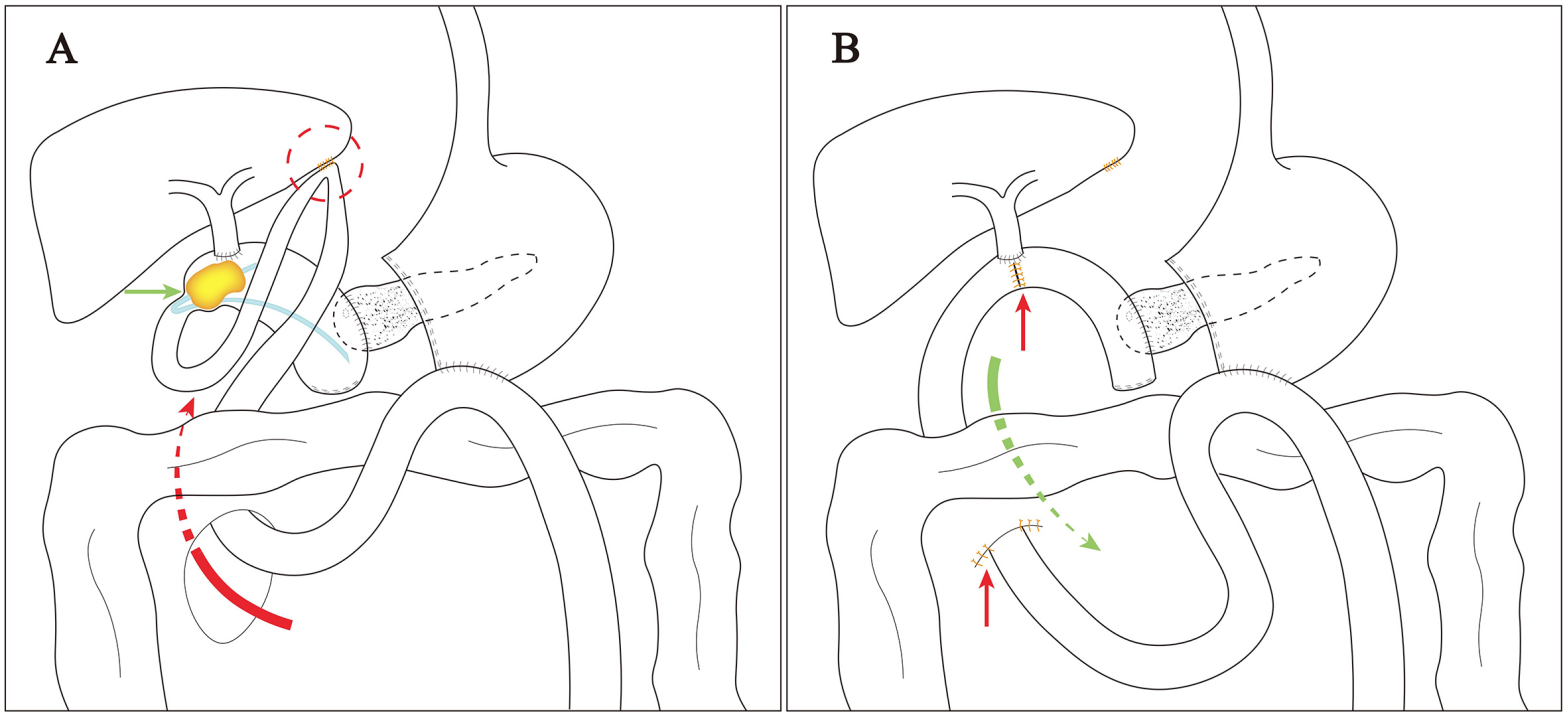
Schematic diagram of intraoperative findings and surgical procedure: **(A)** The excess afferent loop passed through the mesocolic window (red arrow) and adhered to the liver (red circle); ALO induced by gallstone ileus (green arrow); **(B)** pulled back the excess afferent loop (green arrow); sutured mesocolic window and the afferent loop incision (red arrow).

As the patient had unresolved symptoms and a history of open PD, an open operation was performed as planned after the failure of the endoscopic and interventional procedures. Intraoperatively, we observed a redundant afferent loop passing through the mesocolic window and adhering to the liver, forming an acute angle (Figure 3A). After dissecting the adhesions, the structure of the afferent loop was clearly exposed, and it could be seen that the afferent loop was obstructed close to the biliary-enteric anastomosis (Figure 3B). Gallstone ileus was confirmed after longitudinal dissection of the intestine at the site of obstruction (Figures 3C,D). The gallstone removed was approximately 4.5 × 2.5 cm in size and had a low density that allowed it to float on saline (0.9% NaCl). A slender and transparent catheter approximately 15 cm in length was partly wrapped in the gallstone as an “lollipop,” which was consistent with the preoperative imaging (Figure 3F). The afferent loop incision was then closed with the transverse interrupted method using 4–0 Vicryl sutures (Figure 3E). Finally, the redundant afferent loop was pulled back through the mesocolic window and the mesocolic window was closed in the same manner. The intraoperative findings and surgical procedures in this case are shown schematically in Figure 4. The surgery went smoothly, taking a total of 2 h, with blood loss of 50 mL, no blood transfusion required, and no significant intraoperative complications.

Total parenteral nutrition (TPN) was initiated on the second postoperative day. Enteral nutrition was restarted on the fourth postoperative day and TPN was gradually discontinued. Postoperative recovery was uneventful, with no intestinal fistula or abdominal infection. The patient developed an incision infection on the fifth postoperative day and was discharged after approximately two weeks of daily dressing changes. The patient was advised to take ursodeoxycholic acid (UDCA) for at least one year and invited to follow-up at least once per month, no further complications were noted during the 3-month follow-up.

## Discussion

The symptoms of gallstone ileus are usually nonspecific and may include clinical presentations such as abdominal pain, nausea, vomiting and constipation (7). Clinical presentations are often intermittent due to the “tumbling” of the stone in the intestinal tract (8). This could explain why this patient began to suffer from intermittent abdominal pain 2 years ago. Unfortunately, the diagnosis of gallstone ileus was delayed due to lack of further investigations at that time. As the absence of characteristic symptoms, the diagnosis of gallstone ileus is difficult and often requires CT for clarification. In our case, a typical “Mercedes-Benz sign” appears on the CT images due to the presence of gas-containing biliary calculi (9). However, the diagnosis of gallstone ileus can be tricky when the CT images fail to show a gallstone (8). In such cases with typical intermittent symptoms but negative CT images, MRCP may be helpful (10). In our case, ALO induced by gallstone ileus after the Whipple procedure led to intermittent abdominal pain because of the “tumbling” of the stone in the intestinal tract, and also led to obstructive jaundice, biliary tract infection and pancreatitis because of the increased pressure of the afferent loop. We predicted the intraoperative situation before surgery with the help of MRCP and CT, which provided us with a good basis for choosing the surgical plan.

The usual causes of ALO after PD include tumor recurrence, postoperative adhesions, internal herniation, stenosis and foreign bodies (11). Excess small bowel passing through the mesocolic window tends to form adhesion and internal hernia (1). In the current case, internal herniation and adhesions caused a subsequent series of pathological processes that culminated in ALO after PD. The redundant afferent loop passing through the mesenteric window may be twisted and adherent leading to poor bile and pancreatic fluid excretion. Biliary tract infection and pancreatitis may occur in the short term, prolonged poor bile excretion may induce gallstones, and in severe cases, gallstone ileus may occur.

With the advancement of endoscopic technology, management including endoscopic stent (12), drainage (11) and lithotripsy (5) has become feasible in ALO. However, in our case, both endoscopic and interventional treatments failed because of adhesion and internal herniation, making surgical treatment the last option. According to the intraoperative findings, we deduced that bile excretion from the afferent loop was impaired in this patient due to the dual causes of internal herniation and adhesions. For the same reasons, the intra-pancreatic duct catheter used for the pancreatic-enteric anastomosis could not be discharged with intestinal contents and remained in the afferent loop for 8 years, which also became a central cause of stone formation. The stuck catheter may act as an attachment point for stone formation, which, together with the impaired bile excretion, eventually results in the formation of the catheter-centered gallstones.

This patient underwent duct-to-mucosa pancreatojejunostomy during the Whipple procedure 8 years ago, a technique that can be accomplished more easily by placing an intra-pancreatic duct catheter as a stent (13). We have learned some lessons from the current case and concluded that the intra-pancreatic duct catheter serves only as a stent to make the anastomosis easier, and there is no need for excessive length and suture fixation, making it easy to discharge with intestinal contents. Once adhesions develop in the afferent loop, a catheter with excessive length may be difficult to discharge, leading to ALO caused by the foreign body. In addition, we observed that the mesocolic window was too spacious, leading to internal herniation and a series of subsequent problems. Thus, we believe that the afferent loop passing through the mesocolic window needs to be of appropriate length and fixation when using the “trans-mesocolic” route. If the mesocolon is too weak, folded sutures are recommended to strengthen it and avoid internal hernia. Given the infrequency of this condition, the literatures on the management of ALO after the Whipple procedure is limited (14). More clinical reports may help focus attention on the details of the Whipple procedure to prevent ALO.

## Conclusion

Intermittent clinical presentations including abdominal pain, nausea, vomiting and constipation may indicate the presence of gallstone ileus. MRCP and CT may be helpful to confirm the diagnosis and inform the choice of the surgical plan. Internal herniation and adhesions after the Whipple procedure and an intra-pancreatic duct catheter with excessive length can cause a series of pathological processes including gallstone formation and gallstone ileus. Therefore, clinicians should pay attention to the details of the Whipple procedure and remain alert for similar occurrences.

## Patient perspective

I underwent pancreaticoduodenectomy for cholangiocarcinoma 8 years ago, and for a long time after the operation I did not feel any discomfort. However, for the past two years I have experienced recurring abdominal pain. The uselessness of the medication and the frequent episodes of abdominal pain frustrated me until this surgery completely resolved my troubles. I am very grateful to my doctor for his support and guidance during this difficult time. I am now fully recovering and have had no more episodes of abdominal pain, for which I am truly grateful.

## Literature review

A total of 41 relevant articles were retrieved by using the PubMed search system with the keywords “afferent loop obstruction” and “pancreaticoduodenectomy.” Literature types included published case reports, clinical trials and observations. Exclusion criteria included studies with incomplete clinical data and unrelated cases.

## Data Availability

The raw data supporting the conclusions of this article will be made available by the authors, without undue reservation.
